# Treadmill Exercise Training Prevents Myocardial Mechanical Dysfunction Induced by Androgenic-Anabolic Steroid Treatment in Rats

**DOI:** 10.1371/journal.pone.0087106

**Published:** 2014-02-12

**Authors:** Danilo S. Bocalini, Abram Beutel, Cássia T. Bergamaschi, Paulo J. Tucci, Ruy R. Campos

**Affiliations:** 1 Department of Post Graduation in Physical Education, São Judas Tadeu University, São Paulo, Brazil; 2 Cardiovascular Division, Department of Physiology, Federal University of São Paulo, São Paulo, Brazil; 3 Department of Medicine. Cardiology division - Federal University of São Paulo – São Paulo, Brazil; Max-Delbrück Center for Molecular Medicine (MDC), Germany

## Abstract

Elevated concentrations of testosterone and its synthetic analogs may induce changes in cardiovascular function. However, the effects of the combination of anabolic/androgenic steroid (AAS) treatment and exercise training on systolic and diastolic cardiac function are poorly understood. In the present study, we aimed to investigate the effects of low-dose steroid treatment (stanozolol) on cardiac contractile parameters when this steroid treatment was combined with exercise training in rats and the effects of chronic steroid treatment on the Frank-Starling (length-tension curves) relationship. Male Wistar rats were randomly assigned to one of four groups: U (untrained), US (untrained and treated with stanozolol 5 mg/kg/week), T (trained, 16 m/min/1 h) and TS (trained and treated with stanozolol 5 mg/kg/week). Continuous exercise training was conducted 5 days/week for 8 consecutive weeks. The speed of the treadmill was gradually increased to a final setting of 16 m/min/1 h. Experiments were divided into two independent series: 1) central hemodynamic analysis for mean arterial blood pressure (MAP) and cardiac output (CO) measurements and 2) isolated papillary muscle preparation in Krebs solution. Stanozolol treatment significantly increased the MAP and the heart size in untrained and trained rats (U 113±2; T 106±2; US 138±8 and TS 130±7 mmHg). Furthermore, stanozolol significantly decreased developed tension and dT/dt (maximal and minimal) in U rats. However, the developed tension was completely restored by training. The Frank/Starling relationship was impaired in rats treated with stanozolol; however, again, training completely restored diastolic function. Taken together, the present data suggest that AAS treatment is able to decrease cardiac performance (systolic and diastolic functions). The combination of stanozolol and physical training improved cardiac performance, including diastolic and systolic functions, independent of changes in central hemodynamic parameters. Therefore, changes in ventricular myocyte calcium transients may play a cardioprotective role.

## Introduction

Testosterone and its synthetic analogs have been used to increase skeletal muscle mass and enhance physical performance. Furthermore, it is well described that anabolic/androgenic steroids (AAS) have cardiovascular actions and that the heart is a target tissue of AAS, and testosterone is the main physiological hormone in this class [1], [2]. One of the most popular AAS is stanozolol, a 17α-alkylate androgen derivate that exhibits greater anabolic potency (anabolic/androgenic ratio, approximately of 30) and slower hepatic degradation than the natural male hormone [3]. Exogenously administered AAS induce cardiac hypertrophy *in vitro* and *in vivo* [4], [5]. Furthermore, high doses of AAS administered in conjunction with vigorous exercise training in mice leads to cardiac hypertrophy, increased inflammatory cytokine levels and significant stimulation of the sympathetic nervous system [6]. Therefore, the combination of such factors may predispose subjects to myocardial injury. However, the adaptations of cardiac muscle to chronic low-dose AAS exposure and their association with physical exercise are still poorly understood and were the major focus of the present study.

Stanozolol and other AAS are used as adjunct therapies for a variety of medical conditions, e.g., to stimulate erythropoiesis in patients with some anemia conditions, and athletes or even sedentary subjects may use AAS to improve physical performance or appearance [7], [8]. Considering that the anabolic effects of AAS on the cardiovascular system act in both cardiac muscle and arteries, the chronic effects of AAS treatment may induce differential responses in different target organs [9]. Therefore, in the present study, we evaluated the effects of chronic anabolic steroid treatment on isolated papillary cardiac muscle in sedentary and exercise-trained rats. Central hemodynamic function was also evaluated.

In a previous study, Tagarakis et al [10] showed that testosterone impairs the response of cardiac capillary beds to physical exercise. They showed that testosterone treatment profoundly inhibits the exercise-induce augmented capillarization in the heart muscle, whereas under training conditions, it leads to mild myocyte hypertrophy. Others have reported that the association of physical exercise and AAS treatment leads to cardiac fibrosis mediated by activation of angiotensin II type one (AT-1) and mineralocorticoid receptors [11]. However, we have no information regarding the effects of AAS on systolic and diastolic functions in isolated cardiac muscle in combination with the presence or absence of physical exercise training.

It has been reported that higher doses of AAS have deleterious effects on the cardiovascular system [6], [12]. However, to the best of our knowledge, there have been few studies reporting the effects of low-dose steroid treatment combined with exercise training on cardiac function in rats. Thus, the main objectives of the present study were 1) to investigate the effects of low-dose steroid treatment (stanozolol) on cardiac contractile parameters when this steroid treatment was used in combination with exercise training in rats and 2) to investigate the effects of chronic steroid treatment on the Frank-Starling (length-tension curves) relationship.

## Results

**Figure 1:**
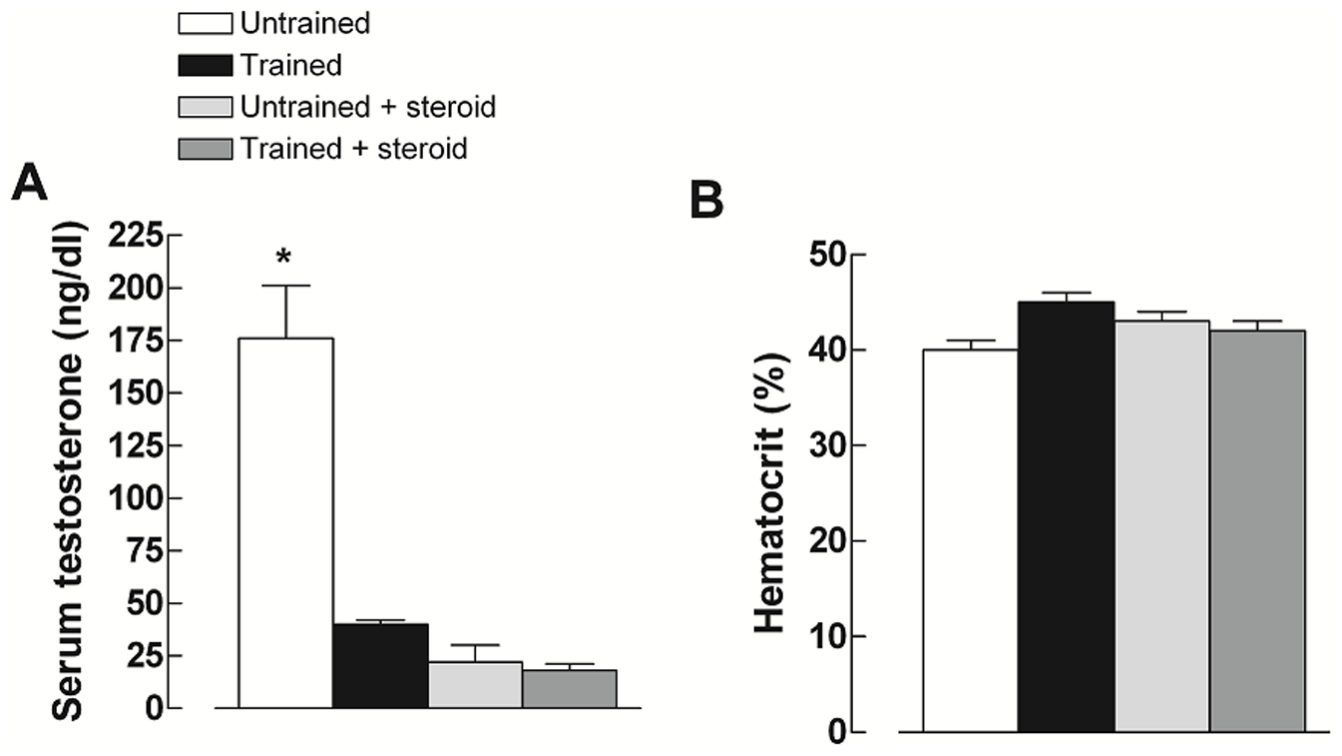
Blood sample biochemical analysis. Panel A: serum testosterone levels in ng/dl. Panel B: hematocrit percentage. Values are expressed at the mean ± SEM for the control (C), low dose (LD) and high dose (HD). * represents significant differences (P<0.05) with respect to other groups as determined by one-way ANOVA–Tukey test.

After eight weeks of steroid treatment, there was no significant difference in body weight among groups (table 1). However, stanozolol treatment and continuous treadmill exercise, both individually and alone, significantly reduced the serum testosterone level compared to that in the U group (U: 176±25, T: 40±2; US: 22±8; TS: 18±3 ng/dl, P<0,05), as shown in figure 1A. Testicle weight was significantly reduced as a consequence of the treatment, as shown in Table 1. There were no differences observed in the hematocrit levels among groups, as shown in figure 1B. Furthermore, there was no difference in right ventricular weight among the groups (table 1). However, significant increases in the left ventricular and total heart weights were found in the US and TS groups compared to their respective untreated groups (table 1).

**Table 1:** Effects on biometric, hemodynamic and contractile parameters by association of anabolic steroid and exercise training.

Variables	U	T	US	TS
*Biometric*				
BW (g)	332±11	299±19	321±21	335±18
TW/BW (mg/g)	5±1	5±1	4±1*	3±1*
RV/BW (mg/g)	0.71±0.11	0.66±0.12	0.66±0.09	0.68±0.04
LV/BW (mg/g)	1.95±0.04	2.08±0.31	2.11±0.19*	2.16±0.16*
HW/BW (mg/g)	2.66±0,17	2.74±0.42	2.77±0.26*	2.85±0.25*
PW (mg)	7.8±0.9	7.8±0.8	7.8±1.3	7.9±0.7
L_max_ (mm)	7.8±1.0	7.8±0.2	7.3±0.5	8.3±0.9
CSA (mm^2^)	1.01±0.11	0.99±0.09	1.06±0.14	0.97±0.14
*Hemodynamic*				
HR (bpm)	363±13	410±21	411±17	372±38
MAP (mmHg)	113±2	106±2	138±8*	130±7*
CI (ml/min/100 g)	38±3	37±5	55±4* #	50±4
TPR (mmHg/ml/min)	0.90±0.07	0.75±0.07	0.80±0.08	0.80±0.12

Values expressed at mean ± SEM of BW: body weight, TW/BW: testicle weight/body weight, RV/BW: right ventricular weight/body weight, LV/BW: left ventricular weight/body weight, HW/BW: heart weight/body weight, PW: papillary weight, L_max_: muscle length papillary muscle length at optimum length, CSA: cross-sectional area, basal levels of HR: heart rate, MAP: arterial pressure, CI: cardiac index and TPR: total peripheral resistance in untrained (U), trained (T), untrained+steroid (US) and trained+steroid (TS).

* represent statistical differences (P<0.05) from U and T.

# represent statistical differences (P<0.05) from TS (by ANOVA–Tukey test).

As observed in table 1, the basal heart rate was not significantly different among groups. However, the basal MAP was higher in both the US and TS groups than in the U and T groups, respectively. The increase in MAP was mediated by an increase in CI. Paradoxically, as shown in table 1, no differences were found in TPR between groups.

**Figure 2:**
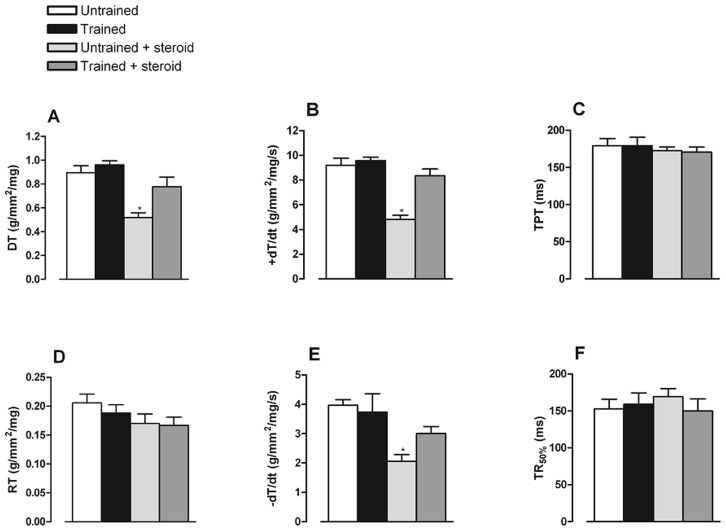
Systolic and diastolic parameters. Values are expressed as the mean ± SEM of developed tension (DT, Panel A), maximum rate of tension development (+dT/dt, Panel B), time to peak tension (TPT, Panel C), rest tension (RT, Panel D), maximum rate of tension decline (−dT/dt, Panel E) and (TR_50%_, Panel F). * represents significant differences (P<0.05) respect to other groups by one-way ANOVA–Tukey test.

No differences were found in Lmax, papillary weight and cross sectional area (Table 1). The systolic and diastolic parameters of myocardial mechanics are shown in figure 2. Significant reductions were found in the US group for developed tension (0.51±0.04 g/mm2/mg), +dT/dt (4.81±0.34 g/mm2/mg/s) and −dT/dt (3.00±0.24 g/mm2/mg/s) relative to other groups: U (DT: 0.89±0.05 g/mm2/mg; +dT/dt: 9.20±0.58 g/mm2/mg/s; −dT/dt: 3.97±0.19 g/mm2/mg/s); T (DT: 0.96±0.03 g/mm2/mg; +dT/dt: 9.57±0.28 g/mm2/mg/s; −dT/dt: 3.74±0.62 g/mm2/mg/s) and TS (DT: 0.77±0.08 g/mm2/mg; +dT/dt: 8.34±0.55 g/mm2/mg/s; −dT/dt: 3.00±0.24 g/mm2/mg/s).

No differences were found in the RT (U: 0.20±0.01 g/mm2/mg, T: 0.18±0.01 g/mm2/mg, US: 0.17±0.01 g/mm2/mg, TS: 0.16±0.01 g/mm2/mg); TPT (U: 179±10 ms, T: 179±12 ms, US: 173±5 ms, TS: 171±7 ms) or RT50% (U: 153±13 ms, T: 159±15 ms, US: 169±11 ms, TS: 150±16 ms), suggesting some preservation of myocardium relaxation, as shown in figure 2.

**Figure 3:**
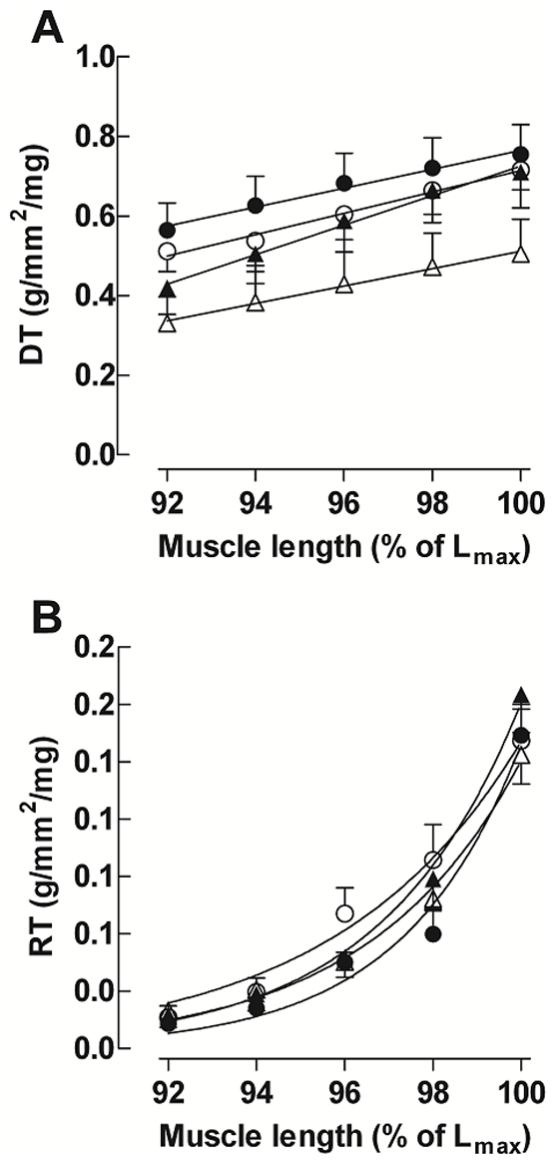
Frank-Starling relationship. Values are expressed as the mean ± SEM of left ventricular papillary muscle developed (A) and resting (B) length-tension curves obtained from untrained (○), trained (•), untrained+steroid (▵) and trained plus steroid (▴) rats. **A**: straight lines were fitted to the developed length-tension relationships using linear regression analysis. **B**: the resting tension-length curves for the four groups were fitted to mono-exponential non-linear relationships.

The Frank-Starling relationship is expressed in g/mm2/mg/%Lmax and is shown in Figure 3A. The slopes for untrained stanozolol-treated rats (US: 0.016±0.004) were different from those of the U (0.032±0.002) and T (0.031±0.002) groups and, curiously, from that of the TS (0.038±0.004) group, suggesting a preservation of the Frank-Starling relationship in TS rats. The resting tension-relationship curves (Figure 3B) did not differ among groups (U: 0.32±0.050; T: 0.43±0.05; US: 0.28±0.02; TS: 0.35±0.02).

## Discussion

The major new findings of the present study were 1) stanozolol treatment increased blood pressure and heart size in rats, 2) stanozolol treatment decreased developed tension and dT/dt (maximal and minimal) in untrained rats, although training completely restored these parameters, and 3) stanozolol treatment impaired the Frank/starling relationship curve, but again, training completely restored diastolic function.

The effects of steroid administration on body mass are conflicting. There are studies showing positive effects [20], no changes or even a reduction in body mass in response to chronic anabolic steroid treatment [21]–[23]. In the present study, no change in body mass index was observed among groups. However, we found a significant increase in cardiac mass in stanozolol-treated rats. A similar result was described previously [23], but there is some controversy regarding this effect [24]. In the present study, the differential effect of stanozolol on the left and right ventricles suggests that the left ventricular hypertrophy was secondary to arterial hypertension because no hypertrophy was found in the right ventricle.

An interesting feature of the efficacy of steroid treatment is the effect of steroids on endogenous levels of testosterone. Previous studies have shown that stanozolol treatment significantly decreases testicular formation in response to testosterone and luteinizing hormone [12], [25]. A significant decrease in plasma levels of testosterone was found in the present study. Interestingly, plasma testosterone levels significantly decreased in response to chronic physical training. A similar result was described previously [26], [27]. However, there are studies showing no changes [28] or even an increase in serum testosterone level in response to physical exercise [29].

A positive correlation has been shown between AAS and arterial hypertension, not only in rats [12], [30] but also in humans [31]. However, the mechanisms underlying hypertension are not fully understood. In the present study, stanozolol treatment increased blood pressure in rats regardless of physical training. The increase in blood pressure was mediated by an increase in cardiac index, and no significant changes were found in TPR, suggesting a preferential effect of stanozolol on cardiac tissue. Training was not able to prevent an increase in blood pressure in response to stanozolol but did lead to a reduction in blood pressure compared to the US group. It has been shown that regular exercise is able to decrease blood pressure in hypertensive subjects [32].

The heart is a target organ for AAS action; there are receptors with a high affinity for testosterone in myocytes [33], [34]. A well-described positive correlation exists between heart hypertrophy and heart failure that is a major independent risk factor for cardiovascular mortality [35]. Furthermore, inflammatory cytokines and excessive stimulation of the sympathetic nervous system are involved in heart hypertrophy [6]. Therefore, the combination of such factors may predispose one to myocardial injury. In the present study, we found that training may protect cardiac function in stanozolol-treated rats.

Finally, in the present study, we found that stanozolol treatment leads to a decrease in cardiac function. However, the combination of stanozolol treatment and physical training improved cardiac performance, including diastolic and systolic functions. The novel finding of the present study is that the improvement in the cardiac performance (systolic and diastolic function) induced by physical exercise in stanozolol-treated rats is apparently independent of changes in central hemodynamic parameters. Therefore, changes in ventricular myocyte calcium transients may play a cardioprotective role.

## Methods

### Animals and treatment

All animal procedures were conducted according to the “Guidelines for Ethical Care of Experimental Animals” and were approved by the Institutional Ethics Committee of the Federal University of São Paulo (UNIFESP) School of Medicine (CEP 0648/04).

The Central Animal House of UNIFESP provided 72 intact male Wistar rats (150–200 g), which were housed in group cages, fed with rat chow and water *ad libitum*, and maintained in a room with constant temperature (23°C) on a 12-h light∶12-h dark cycle. The animals were randomly assigned to one of four groups: U (untrained), US (untrained and treated with stanozolol 5 mg/kg/week), T (trained, 16 m/min/1 h), and TS (trained and with stanozolol 5 mg/kg/week. Stanozolol was administered intramuscularly, and the same volume of saline was injected into the control groups, as previously described [12]. All rats were familiarized with treadmill running. Continuous exercise training was conducted for 60 min 5 days/week for 8 consecutive weeks during the light period of the light-dark cycle. The speed of the treadmill was gradually increased to a final setting of 16 m/min. The exercise training protocol was based on a previous study [13].

### Biochemical analysis

Blood samples were collected via a venous catheter in the four groups of animals, and the serum levels of testosterone were quantified by radioimmunoassay, using standard commercially available kits.

### Hemodynamic analysis

The hemodynamic parameters were measured in conscious rats using a Cardiomax III thermodilution system (Columbus Instruments Inc., Ohio, USA), as described in previous studies [14]. In this series of experiments, the animals were instrumented for hemodynamic measurements at least 24 hours before the experiments under halothane anesthesia (2%).

### Isolated papillary muscle mechanics

Immediately after echocardiogram, the heart was quickly removed and placed in oxygenated Krebs solution. As previously described by our laboratory [14]–[18], the papillary muscle was dissected carefully from the left ventricle, mounted between two spring clips and placed vertically in a chamber containing Krebs solution (28°C) oxygenated with 100% O2 at a pH of 7.40±0.02. The composition of the Krebs solution was as follows (mmol/L): NaCl 132; KCl 4.69; CaCl2 1.5; MgSO4 1.16; KH2PO4 1.18; C6H12O6 5.50; HEPES 20. The lower spring clip was attached to the bottom of the chamber, and the upper spring clip was connected by a thin steel wire to an isometric transducer (model FT03E; Grass Instrument, Quincy, MA, USA) connected to a micrometer for adjustment of muscle length. Preparations were stimulated 12 times/min with 5-ms square-wave pulses through parallel platinum electrodes at voltages that were approximately 10% greater than the minimum stimulus required to produce a maximal mechanical response. After a 60-minute equilibration period, during which preparations were permitted to contract isotonically under light loading conditions (0.4 g), papillary muscles were loaded to contract isometrically for 15 min and stretched to the apices of their length–tension curves (Lmax). The mechanical behavior of the papillary muscles was evaluated under basal conditions, as well as at 98, 96, 94 and 92% of Lmax, allowing for determination of Frank–Starling curves as described previously [15]–[16]. The following parameters were measured during isometric contractions: peak developed tension (DT), resting tension (RT), maximum rate of tension development (+d*T*/d*t*) and tension decline (−d*T*/d*t*), time to peak tension (TPT) and time from peak tension to 50% relaxation (RT50%). At the end of each experiment, muscle length at *L*max was measured, and the muscle between the two clips was blotted dry and weighed. The muscle cross-sectional area (CSA) was calculated from the muscle weight and length by assuming cylindrical uniformity and a specific gravity of 1. The values were normalized to conform to previous publications [15]–[19]. After the papillary muscle was removed, left and right ventricles were separated and weighed for posterior evaluation of biometrical parameters.

### Statistical analysis

The data were expressed as the mean ± SEM. One-way ANOVA was used for the group comparisons followed by a post hoc Tukey's test. The DT–length relations were evaluated by linear regression analysis, and the slopes were compared by one-way ANOVA. The RT–length curves were fit to a monoexponential relation, as follows: y = β0e^β1x^, where β0 and β1 are the constants of the curve. These non-linear relationships were compared between groups by constant stiffness values. Statistical analyses were performed with Prism software (version 4.0, San Diego, CA, USA). Values of *P*<0.05 were considered statistically significant.
